# Opponent familiarity and contest experience jointly influence contest decisions in *Kryptolebias marmoratus*

**DOI:** 10.1186/s12983-014-0092-7

**Published:** 2014-12-10

**Authors:** Cheng-Yu Li, Yusan Yang, Pey-Yi Lee, Yuying Hsu

**Affiliations:** Department of Life Science, National Taiwan Normal University, No. 88, Section 4, Ting-Chou Rd, Taipei, 11677 Taiwan

**Keywords:** Animal contest, Information, Winner-loser effect, Individual recognition, Familiarity, *Kryptolebias marmoratus*

## Abstract

**Introduction:**

Individual recognition and winner/loser effects both play important roles in animal contests, but how their influences are integrated to affect an individual’s contest decisions in combination remains unclear. Individual recognition provides an animal with relatively precise information about its ability to defeat conspecifics that it has fought previously. Winner/loser effects, conversely, rely on sampling information about how an animal’s ability to win compares with those of others in the population. The less precise information causing winner/loser effects should therefore be more useful to an individual facing an unfamiliar opponent. In this study, we used *Kryptolebias marmoratus*, a hermaphroditic mangrove killifish, to test whether winner/loser effects do depend on opponent familiarity. In addition, as previous studies have shown that subordinates that behave aggressively sometimes suffer post-retreat retaliation from contest winners, we also explored this aspect of contest interaction in *K. marmoratus*.

**Results:**

In the early stages of a contest, subordinates facing an unfamiliar dominant were more likely to signal their aggressiveness with either gill displays or attacks rather than retreating immediately. A winning experience then increased the likelihood that the most aggressive behavioral pattern the subordinates exhibited would be attacks rather than gill displays, irrespective of their opponents’ familiarity. Dominants that received a losing experience and faced an unfamiliar opponent were less likely than others to launch attacks directly. And subordinates that challenged dominants with more aggressive tactics but still lost received more post-retreat attacks from their dominant opponents.

**Conclusions:**

Subordinates’ contest decisions were influenced by both their contest experience and the familiarity of their opponents, but these influences appeared at different stages of a contest and did not interact significantly with each other. The influence of a losing experience on dominants’ contest decisions, however, did depend on their subordinate opponents’ familiarity. Subordinates and dominants thus appeared to integrate information from the familiarity of their opponents and the outcome of previous contests differently, which warrants further investigation. The higher costs that dominants imposed on subordinates that behaved more aggressively toward them may have been to deter them from either fighting back or challenging them in the future.

## Introduction

By taking part in contests, animals expend energy and time, risk physical injuries and predation [1,2] and forgo other opportunities (for instance, the time and energy could be used to search for alternative resources). Since the more able contestant has the better chance of winning, resolving a contest quickly and avoiding injury [3,4], an individual’s potential contest cost should decrease with its fighting ability and increase with its opponent’s [5]. It therefore benefits animals to pay attention to information about their and their opponents’ fighting ability [6], information that may be acquired from various sources. The outcomes of previous contests, for instance, can provide individuals with sampling information about how their fighting ability compares with those of others in the population; winning experiences raise while losing experiences lower individuals’ estimates of their own fighting abilities [7-9] but see [10]. These changes in estimated fighting ability modify individuals’ anticipated fighting costs and therefore their contest decisions. Pairs of contestants that have fought previously can also recognize each other and use the outcome of their previous interaction to settle their future conflicts (individual recognition [11-13]). By not fighting opponents to which it has recently lost, an individual can avoid the unnecessary costs of contests that it has a very low chance of winning.

Both winner/loser effects [14] and individual recognition [11] have been demonstrated to influence the contest decisions of individuals in a wide array of taxa including insects, arachnids, crustaceans, fish, reptiles, birds and mammals. Individuals tend to be more aggressive in the period following a win: they are more likely to initiate contests and retaliate when provoked; they tend to persist longer before retreating and have a higher probability of winning their next contest (winner effect) [15-18]. The converse is true after a loss, when individuals tend to become more passive and are likely to retreat sooner when challenged (loser effect) [19-23]. Subordinates exposed to their former (familiar) dominants often have more pronounced avoidance responses, and retreat again more readily than when paired up with unfamiliar dominants [24-26].

Both individual recognition and winner/loser effects involve animals using information from previous contests while making subsequent contest decisions, but to date there is no published research on how the two sources of information jointly influence contest decisions and outcomes. Individual recognition provides an individual with relatively precise information on how its ability to win compares with its previous opponents’. By contrast, winner/loser effects rely on highly imprecise sampling information about how an animal’s ability to win compares with those of the population in general. Winner/loser effects should therefore be more apparent in fights between unfamiliar opponents (when more precise information is not available) and less so to fights between familiar opponents (which should be settled based on the more reliable information from the two opponents’ previous interactions).

In this study, we tested whether the importance of winning/losing experiences to contest decisions does indeed depend on the familiarity of the opponent, using *Kryptolebias marmoratus*, a hermaphroditic mangrove killifish, as the study organism. This fish’s contest decisions are sensitive to contest costs - smaller individuals and those facing larger competitors tending to retreat sooner without escalating fights into mutual attacks [27]. The fish also exhibits winner and loser effects in contests [21,28] and is capable of distinguishing between kin (propagated from the same parental fish) and non-kin (individuals of different lineages) and between familiar and unfamiliar kin [29]. Building on these findings, we investigated whether winner/loser effects are more prominent in contests between unfamiliar than between familiar contestants.

**Figure 1:**
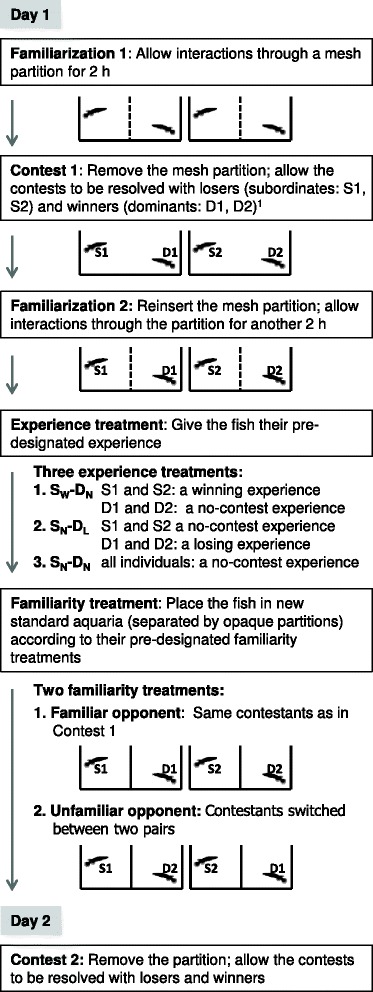
**Experimental procedures.** A diagram showing the experimental procedures. ^1^The winner and the loser from Contest 1 are referred to as the dominant and the subordinate opponent of a contest pair, respectively, throughout the study.

This study used a 2 (familiarity treatments: familiar or unfamiliar opponent) × 3 (experience treatments) factorial design. The experimental procedures consisted of six steps: Familiarization 1, Contest 1, Familiarization 2, Experience treatment, Familiarity treatment and Contest 2 (summarized in Figure 1). On Day 1, two size-matched individuals were allowed to interact with each other through a clear-mesh partition for two hours (Familiarization 1). The mesh partition was then removed to allow the two individuals to fight until the fight was resolved with a clear winner and loser (Contest 1). The winner and the loser from Contest 1 were denoted as the dominant and the subordinate opponent of a contest pair, respectively, throughout the study. The mesh partition was reinserted to separate the fish after Contest 1 was resolved. The two individuals of a contest pair were allowed to interact through the mesh partition for another two hours (Familiarization 2). Afterwards, the subordinate and the dominant individuals received a pre-designated type of experience training (Experience treatment). Three experience treatments were used: (1) giving the subordinate a winning experience and its dominant opponent in Contest 2 a no-contest experience (S_W_-D_N_ treatment), (2) giving the subordinate a no-contest experience and its dominant opponent a losing experience (S_N_-D_L_ treatment) and (3) giving both contestants a no-contest experience (S_N_-D_N_ treatment). These three experience treatments allowed us to evaluate winner effects in the subordinates and loser effects in the dominants. We did not attempt to evaluate winner effects in the dominants or loser effects in the subordinates; these fish were expected to exhibit very high/low levels of aggression because of their good/poor fighting ability and their winning/losing experience in Contest 1, so that any increase/decrease in aggressiveness caused by an additional winning/losing experience would have been hard to measure. After the experience treatment, the fish were assigned into pairs again (Familiarity treatment). Half of the fish were paired up with the same opponents they had faced in Contest 1 (familiar opponent treatment, F). The other half of the subordinates faced different sized-matched dominants, and the dominants faced different size-matched subordinates (unfamiliar opponent treatment, UF) (adopted from D’Ettorre & Heinze [26]). Contest 2 (between familiar or unfamiliar opponents) was then staged on the next day (Day 2).

The behavior of subordinates and dominants in Contest 2 was used to examine the influence of familiarity and contest experience. *K. marmoratus*, like many other fish [30-33], uses head-on opercular displays at early stages of a contest [27]. If both opponents persist after mutual displays, one of them will launch an attack. Mutual attacks can cause physical injuries and have also been shown to use more energy [1,34,35] and are thus more costly and risky than mutual displays. In *K. marmoratus*, attacks are usually preceded by mutual opercular displays, but some individuals show their readiness to invest in high-cost interactions without the information from mutual displays by launching attacks directly [22]. We consider this group of individuals to be the most aggressive (Level 4 = attacking directly), followed by those that launch attacks after exhibiting opercular displays (Level 3 = attacking after displaying) and then by those that only exhibit opercular displays (Level 2 = displaying). Individuals that do not exhibit opercular displays or launch attacks are non-aggressive (Level 1 = none). We tested whether the effect of a winning experience on the subordinates’ aggressiveness and the effect of a losing experience on the dominants’ aggressiveness were dependent on the familiarity of their opponents, by examining the most aggressive behavior they exhibited in Contest 2.

As well as expending more energy and risking more injury during a contest [1,34,35], subordinates behaving aggressively may also suffer post-retreat retaliation by victorious dominants. In serins (*Serinus serinus*), for instance, subordinates that initiated agonistic interactions with dominants with attacks (instead of displays) but lost were more likely to receive post-retreat attacks (instead of displays) from the dominants [36]. We investigated this aspect of *K. marmoratus*’s contest behavior by analyzing the number of attacks that the winners delivered to the losers after they had retreated in Contest 2.

## Results

### The influence of familiarity and contest experience on subordinates’ and dominants’ behaviors in Contest 2

**Figure 2:**
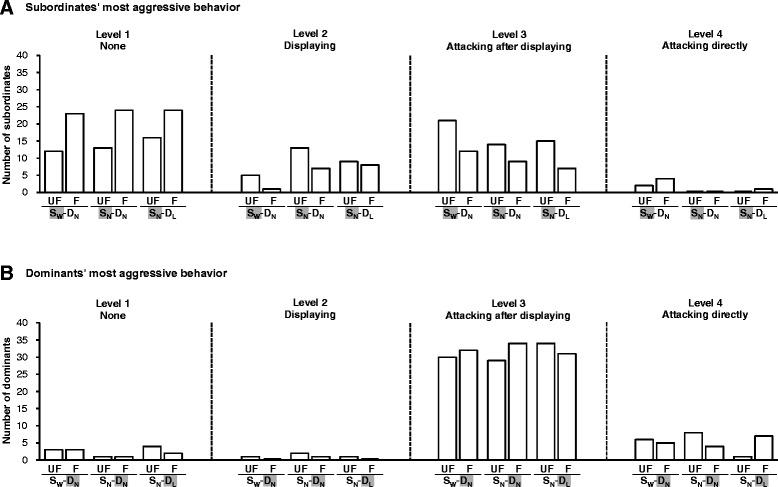
**Subordinates’ and dominants’ most aggressive behavior in Contest 2.** The numbers of **(A)** subordinates and **(B)** dominants for which the most aggressive behavior in contest 2 was no aggressive behaviors (Level 1), exhibiting opercular displays (Level 2), launching attacks after having exhibited opercular displays (Level 3) or launching attacks directly without displaying first (Level 4), broken down by different combinations of familiarity (UF: unfamiliar; F: familiar) and experience (S_W_-D_N_, S_N_-D_N_ and S_N_-D_L_) treatments.

A total of 240 Contest 2s were staged (between 480 individuals). A large proportion (112/240 = 46.7%) of the subordinates behaved non-aggressively (Level 1) in Contest 2, and neither exhibited opercular displays nor launched attacks. The most aggressive behavior exhibited by the remaining subordinates was displaying (Level 2) in 43 cases (17.9%), attacking after displaying (Level 3) in 78 cases (32.5%) and attacking directly (Level 4) in 7 cases (2.9%) (Figure 2A). Because only 7 out of the 240 subordinates exhibited the Level-4 behavior (launched attacks directly) in Contest 2, we pooled them with the individuals exhibiting Level-3 behavior (launched attacks after displaying) to form an “attacking” group. Consequently, the subordinates were re-classified into 3 groups for subsequent analyses: none (Level 1), displaying (Level 2) and attacking (Levels 3 and 4).

The dominants, conversely, behaved aggressively (Figure 2B). Very few of them either failed to display any aggressive behaviors (Level 1: 14/240 = 5.8%) or exhibited only displays (Level 2: 5/240 = 2.1%). The vast majority of them either launched attacks after displaying (Level 3: 190/240 = 79.2%) or launched attacks directly (Level 4: 31/240 = 12.9%). Because very few of the dominants exhibited Level-1 and Level-2 aggressive behaviors, we pooled them with the Level-3 individuals. Consequently, the dominants were divided into only two groups for subsequent analyses: those that did not launch attacks directly (Levels 1 to 3) and those that did (Level 4).

### On subordinates’ behavior

We first examined the influence of familiarity and contest experience on the relative likelihood that the subordinates would show some aggression (either displaying or launching attacks, Levels 2–4; Figure 2A: 2nd – 4th panels combined) rather than no aggression (Level 1; Figure 2A: 1st panel) (Table 1(1)). There was no significant interaction between the familiarity and the experience treatments (*P* = 0.356) and no significant effects of the experience treatments (*P* = 0.462) on this relative tendency. The effect of the familiarity treatment, however, was significant (*P* < 0.001); subordinates fighting unfamiliar dominants were more likely either to exhibit displays or to launch attacks than those fighting familiar dominants. Moreover, subordinates whose dominant opponents launched attacks directly tended to behave more submissively, and were less likely either to exhibit displays or to launch attacks (*P* < 0.001).

**Table 1:** **The influence of familiarity and experience treatments on subordinates’ strategies in Contest 2**

		**(1) Displaying/attacking**			**(2) Attacking**		
		**vs.**			**vs.**		
		**Nonaggressive**			**Displaying**		
		**(** ***N*** **= 240)**			**(** ***N*** **= 128)**		
***Effect***	***df***	**b ±SE**	***χ*** ^***2***^	***P***	**b±SE**	***χ*** ^***2***^	***P***
Familiarity (UF vs. F)	1	1.20±0.30	16.35	<0.001*	−0.26±0.50	0.27	0.607
Experience	2		1.54	0.462		9.91	0.007*
S_W_-D_N_ vs. S_N_-D_N_	1	0.06±0.37	0.03	0.872	2.05±0.68	9.15	0.003*
S_N_-D_L_ vs. S_N_-D_N_	1	−0.35±0.36	0.96	0.327	0.11±0.46	0.06	0.810
Familiarity × experience	2		2.07	0.356		1.91	0.385
Dominant attacking directly (Y vs. N)	1	−2.31±0.58	16.00	<0.001*	−0.03±1.36	0.00	0.980
Size	1	−0.08±0.06	1.41	0.236	−0.05±0.10	0.20	0.657
Lineage	4		11.36	0.023*		0.70	0.952

Generalized linear models evaluating the influence of familiarity (F: familiar; UF: unfamiliar) and experience (S_W_-D_N_: subordinates received a winning experience and dominants received a no-contest experience; S_N_-D_L_: subordinates received a no-contest experience and dominants received a losing experience; S_N_-D_N_: both opponents received a no-contest experience) treatments as well as the interaction between them on the most aggressive behavior the subordinates exhibited in Contest 2. Because very few subordinates attacked without displaying first (Level 4), these individuals were pooled with those that attacked after first displaying (Level 3) to form an “attacking” group. Consequently, the subordinates were classified into 3 groups based on the most aggressive behavior exhibited in Contest 2: nonaggressive, displaying and attacking. We examined (1) the likelihood that subordinates would act aggressively (by either exhibiting opercular displays or launching attacks toward their dominant opponents; Levels 2–4) compared with the likelihood of them not acting aggressively (exhibiting none of these behaviors; Level 1) and (2) for the aggressive subordinates (Levels 2–4) only, the likelihood that they would launch attacks (Levels 3 and 4) compared with the likelihood of going no further than exhibiting opercular displays (Level 2). The pair’s body size and lineage and whether the dominant opponent’s launched attacks directly (Y: yes; N: no) were included in the models to account for their influences. Contrast analyses were used to evaluate the differences between the effects of different levels of a treatment. (χ^2^: Ward χ^2^; *: *P* < 0.05).

We then excluded the subordinates that showed no aggressive behavior (Level 1), and examined how likely the remaining subordinates (Levels 2–4, *N* = 128) were to launch attacks (Levels 3 and 4) rather than just to exhibit opercular displays and go no further (Level 2) (Table 1(2)). Experience was the only factor that had a significant effect on this relative tendency (*P* = 0.007). Post-hoc tests showed that the subordinates’ winning experience (*P* = 0.003), but not their dominant opponents’ losing experience (*P* = 0.810), significantly increased the relative tendency of the subordinates to launch attacks.

### On dominants’ behavior

**Figure 3:**
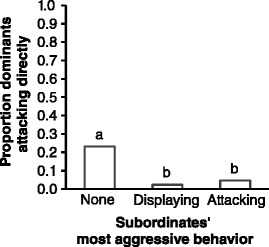
**Association between dominants’ tendency to launch attacks directly and their subordinate opponents’ aggressiveness.** The proportion of dominants that launched attacks directly in Contest 2 when fighting with the subordinate opponents that exhibited different levels of aggression. ‘None’ – subordinate did not display at or attack the dominant (N = 112), ‘Displaying’ – the most aggressive behavior exhibited was opercular display (N = 43), ‘Attacking’ – the most aggressive behavior exhibited was an attack (N = 85). Bars labeled with different letters are significantly different at *P* < 0.05 (contrast analyses).

There was a significant interaction between the familiarity and the experience treatments on the dominants’ tendency to launch attacks directly (Level 4; Figure 2B: 4th panel) (*P* = 0.018; Table 2). Post-hoc analyses showed that dominants’ losing experience significantly decreased the dominants’ tendency to launch attacks directly only when they were facing unfamiliar opponents (*P* = 0.010) and not when they were facing familiar opponents (*P* = 0.212). Their subordinate opponents’ winning experiences did not significantly influence the dominants’ tendency to launch attacks directly for either familiarity treatment (P ≥ 0.400). Furthermore, the tendency of the dominants to launch attacks directly was significantly associated with the aggressiveness of their subordinate opponents (*P* < 0.001; Figure 3): dominants were most likely to launch attacks directly when their subordinates exhibited no aggressive behavior.

**Table 2:** **Influence of familiarity and experience treatments on dominants’ strategies in Contest 2**

		**Attacking directly**		
		**vs.**		
		**Behaving less aggressive**		
		**(** ***N*** **= 240)**		
***Effect***	***df***	**b ±SE**	***χ*** ^***2***^	***P***
Familiarity (UF vs. F)	1	0.09±0.54	0.03	0.871
Experience	2		3.52	0.172
S_W_-D_N_ vs. S_N_-D_N_	1	0.24±0.56	0.20	0.657
S_N_-D_L_ vs. S_N_-D_N_	1	−1.08±0.71	2.36	0.125
Familiarity × experience	2		8.02	0.018*
UF: S_W_-D_N_ vs. S_N_-D_N_	1	−0.19±0.74	0.06	0.800
S_N_-D_L_ vs. S_N_-D_N_	1	−3.11±1.21	6.65	0.010*
F: S_W_-D_N_ vs. S_N_-D_N_	1	0.68±0.81	0.71	0.400
S_N_-D_L_ vs. S_N_-D_N_	1	0.95±0.76	1.56	0.212
Subordinate’s aggressive behavior	2		16.11	<0.001*
Size	1	0.28±0.09	9.09	0.003*
Lineage	4		11.86	0.018*

Generalized linear model evaluating the influence of familiarity (F: familiar; UF: unfamiliar) and experience (S_W_-D_N_, S_N_-D_L_ and S_N_-D_N_) treatments as well as the interaction between them on the tendency of the dominants to launch attacks directly (Level 4 vs. Levels 1 to 3) in Contest 2. The pair’s body size and lineage and the most aggressive behavior the subordinate exhibited (none, displaying or attacking) were included in the model to account for their influences. Contrast analyses were used to evaluate the differences between the effects of different levels of a treatment. Because the interaction between familiarity and experience treatments was significant, contrast analyses were also conducted to evaluate the effect of experience treatments in unfamiliar and familiar opponent treatment separately. (χ^2^: Ward χ^2^; *: *P* < 0.05).

### On contest outcome

**Figure 4:**
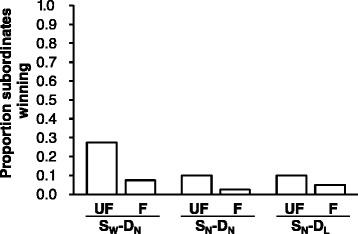
**Proportion of Contest 2s won by subordinates.** The proportion of Contest 2s won by the subordinates subjected to different combinations of familiarity (UF: unfamiliar; F: familiar) and experience (S_W_-D_N_, S_N_-D_N_ and S_N_-D_L_) treatments.

Dominants won the majority (215/240 = 89.6%) of Contest 2s as expected; subordinates only won 25 of them (Figure 4). The familiarity treatment was the only factor that significantly influenced contest outcome (*P* = 0.017; Table 3); subordinates were more likely to win in Contest 2 if fighting an unfamiliar dominant opponent.

**Table 3:** **Influence of familiarity and experience treatments on the outcome of Contest 2**

		**Subordinates won**		
		**vs.**		
		**Subordinates lost**		
		**(** ***N*** **= 240)**		
***Effect***	***df***	**b ±SE**	***χ*** ^***2***^	***P***
Familiarity (UF vs. F)	1	1.29±0.54	5.67	0.017*
Experience	2		3.74	0.154
S_W_-D_N_ vs. S_N_-D_N_	1	1.16±0.68	2.93	0.087
S_N_-D_L_ vs. S_N_-D_N_	1	0.34±0.73	0.22	0.638
Familiarity × experience	2		0.60	0.740
Size	1	−0.13±0.10	1.55	0.213
Lineage	4		3.91	0.419

Generalized linear model evaluating the influence of familiarity (F: familiar; UF: unfamiliar) and experience (S_W_-D_N_, S_N_-D_L_ and S_N_-D_N_) treatments as well as the interaction between them on whether or not the subordinate won Contest 2. The pair’s body size and lineage were included in the model to account for their influences. Contrast analyses were used to evaluate the differences between the effects of different levels of a treatment. (χ^2^: Ward χ^2^; *: *P* < 0.05).

### Influence of familiarity and contest experience on post-retreat aggression in Contest 2

**Figure 5:**
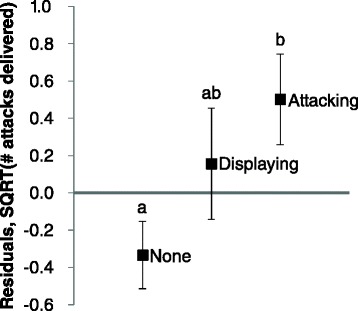
**Post-retreat attacks that victorious dominants delivered to subordinates after Contest 2.** The number of post-retreat attacks (square root transformed) that the victorious dominants (N = 215) delivered to the subordinates that exhibited different levels of aggression toward them after Contest 2: ‘None’ – subordinate did not display at or attack the dominant (N = 112), ‘Displaying’ – the most aggressive behavior exhibited was opercular display (N = 41), ‘Attacking’ – the most aggressive behavior exhibited was an attack (N = 62). Values presented are the residuals (mean ± SE) from a multiple regression model of the influence of opponent type and experience on the number of post-retreat attacks, controlling for body size and lineage. Means labeled with different letters are significantly different at *P* < 0.05 (Tukey multiple comparisons).

The number of attacks (square root transformed; mean ± SE) delivered by the winners to the losers of Contest 2s after they had retreated did not differ significantly (*t*_238_ = 0.431, *P* = 0.667) between dominant (3.81 ± 0.14) and subordinate winners (3.99 ± 0.41). We first used a general linear model to examine the effects of familiarity and contest experience on the number of attacks that victorious dominants delivered to their subordinate opponents (*N* = 215) after they had retreated. None of the factors examined, including familiarity (*F*_*1,204*_ = 0.95, *P* = 0.330), experience (*F*_*2,204*_ = 0.83, *P* = 0.437), the interaction between them (*F*_*2,204*_ = 0.32, *P* = 0.727) and the contest pairs’ size (*F*_*1,204*_ = 1.97, *P* = 0.162) and lineage (*F*_*4,204*_ = 1.90, *P* = 0.111), had any significant relationship with the number of post-retreat attacks. We then used the residuals from the model to test whether victorious dominants delivered different number of post-retreat attacks to subordinates that had exhibited different levels of aggressiveness. Victorious dominants delivered significantly different numbers of post-retreat attacks to the subordinates that had exhibited different aggressive behaviors (*F*_2,212_ = 3.97, *P* = 0.020; Figure 5). Subordinates that had behaved more aggressively toward the dominants tended to receive higher numbers of post-retreat attacks from the dominants, although only the difference in the number of attacks received between the subordinates that did not show any aggressive behavior and those that launched attacks reached the significance level (*P* = 0.017, Tukey multiple comparisons).

We did not analyze whether the number of post-retreat attacks delivered by the 25 subordinates that won Contest 2 to their defeated dominant opponents was influenced by either the experimental treatments (because the sample size was too small to yield meaningful conclusions) or their opponents’ aggressiveness (as all defeated dominants had been aggressive and launched attacks).

## Discussion

The objective of our study was to test the hypothesis that a previous winning or losing experience would have less effect on an individual’s contest behavior when it faced a familiar rather than an unfamiliar opponent, using *K. marmoratus* as the study organism. For the dominants it did: the effect of a losing experience was only visible in dominants that faced unfamiliar opponents, and in these cases, individuals were less likely to exhibit the most aggressive form of behavior (attacking directly) than the others. Both a winning experience and the familiarity of their opponents influenced subordinates’ contest behavior, but their effects appeared at different stages of the contest, and the effect of a winning experience was not dependent on the familiarity of the opponent. Subordinates were more likely to exhibit some form of aggression to unfamiliar than to familiar dominants irrespective of the experience treatment they received. And, of the subordinates that exhibited some form of aggression, those that had received a winning experience were more likely to attack their opponents than those that had received a no-contest experience, regardless of their opponent’s familiarity. Familiarity and experience treatments had relatively little influence on the outcome of Contest 2 despite their significant influences on both the subordinates’ and the dominants’ behaviors in the contests. As the dominant opponents won the majority (89.6%) of Contest 2s, contest outcome was primarily determined by the contestants’ intrinsic fighting ability. In addition to the familiarity and experience treatments, the fish’s contest decisions were also influenced by its opponent’s behavior in the contest, indicating that the two contestants paid close attention and responded to each other’s behavior. The negative associations between the subordinate and the dominant opponent’s level of aggression suggested that individuals tended to behave more cautiously (i.e., less aggressively) when interacting with aggressive opponents.

We had hypothesized that information from a winning or losing experience would be more valuable to individuals that could not draw on more reliable information arising from having faced the same opponent before. Information is useful to an individual because it reduces uncertainty in the individual’s decision-making [37]. Prior experience can provide animals with information about their internal and external circumstances, enabling them to evaluate costs and/or benefits associated with different behavioral options more accurately [37-39]. Therefore, the fact that a winning experience influenced a subordinate’s behavior, even in a contest with a familiar dominant, implies some remaining uncertainty about the relative abilities of the contestants in that pairing and the outcomes of their future contests. The result that 6 out of the 120 (5%) Contest 2s between familiar opponents were won by subordinates confirms this. Furthermore, the winning experience in this study was imposed on the subordinates after they had been defeated by the dominants in Contest 1, and, as a more recent event providing more up-to-date information, should have been expected to carry a higher value. Individuals of *K. marmoratus* place a higher value on information from a more recent event than on an older one. For instance, when the fish were given two contest experiences, both experiences affected the fish’s contest behaviors, but the more recent experience had the greater effect [21,22]. If both individual recognition and contest experience provide subordinates with information about their relative fighting ability as hypothesized, the information arising from the winning experience could have been used to update the information arising from the first defeat and could have caused the subordinates to exhibit a winner effect even in contests with familiar dominants.

It is not entirely clear why the effect of losing on the dominants’ tendency to attack directly (without displaying first) was dependent on the familiarity of the opponent while the effect of winning on the subordinates’ tendency to attack (with or without displaying first) was not. Recent studies have revealed that the fish’s responsiveness to the influence of recent contest experience depends on both its hormonal state [40] and a contest-experience treatment imposed on it one-month previously [41]. Individuals with lower levels of cortisol, testosterone and 11-ketotestosterone, a state that corresponds to subordination and a reduced likelihood of winning [40,42,43], showed significant loser effects whereas those with higher levels of the hormones did not [40]. Similarly, individuals that were given a forced losing experience one month before a second experience-training session were more likely to exhibit both winner and loser effects than those given a forced winning experience at the same time. These results suggest that individuals with or perceiving themselves to have low fighting ability are more responsive to the information derived from recent contest experiences than those with or perceiving themselves to have good fighting ability. As the losers and winners of Contest 1, the subordinates and the dominants of the present study demonstrated poor and good intrinsic fighting ability, respectively, and were probably more and less responsive, respectively, to the influence of a winning or losing experiences. Therefore, it could be that the experience effect was only detectable in dominants exposed to an unfavorable situation (i.e., fighting an unfamiliar subordinate) and not in those exposed to a favorable situation (i.e., fighting a familiar subordinate) simply because they were dominants. Subordinates, on the other hand, readily responded to contest experience, irrespective of the familiarity of their opponents. Another possibility is that the difference in the behavioral trends between the dominants and the subordinates resulted, at least partially, from different behavioral measures which it was necessary to use for the two groups of individuals.

The number of the post-retreat attacks that the dominants delivered to the subordinates in Contest 2 did not depend on familiarity or contest experience, but did depend on the subordinates’ contest strategies. Dominant winners of Contest 2 responded more aggressively toward more aggressive subordinate losers, consistent with the trends observed in serins [36]. Higher energy costs are associated with attacks than with displays [35], and dominants could incur higher energetic costs by attacking subordinates more often [34]. Some function should therefore accompany the heightened aggression of provoked dominants to compensate for the costs. Subordinates that challenge their dominant opponents are probably more aggressive than those that do not, and might only be discouraged from fighting back by heightened aggression or more costly retaliation. The dominants’ costly revenge strategy could also reduce the same or other subordinates’ willingness to challenge them in the future, especially in populations in which individuals encounter each other repeatedly and are capable of recognizing their previous opponents. Although this possibility has not been examined in *K. marmoratus,* there is empirical evidence that individuals of other species avoid fighting opponents that they have previously observed to behave aggressively. For instance, bystanders of green swordtail (*Xiphophorus helleri*) exhibited a tendency not to initiate aggressive interactions with individuals that they had previously observed to escalate fights with a 3rd party [44].

Our study used individuals from five isogenic lineages of *K. marmoratus*. Our results on the effect of familiarity coincide with the conclusions of a previous study of the fish [29], which used individuals of different lineages from us and found that the fish of each lineage could distinguish between familiar and unfamiliar conspecifics of the same lineage. Similarly, two different lineages used in a previous study [21,22] also exhibited winner and loser effects. It thus appears that the tendency to behave differently toward familiar and unfamiliar individuals of the same lineage and the tendency to modify contest behavior after winning and losing experiences are common traits to different lineages of this fish.

It is not clear what types of sensory cues enabled the subordinate individuals of *K. marmoratus* to respond differently to familiar and unfamiliar dominants, as the fish in the study could exchange both visual and chemical signals. A previous study of *K. marmoratus*, however, demonstrated that olfaction alone was enough for hermaphrodites to distinguish hermaphrodites from males or for males to distinguish hermaphrodites of their own lineage from those of a different lineage [45]. As the water in the fish’s mangrove habitat is often highly turbid (because of the high microbial productivity and microalgae) [46], olfactory signals could enable weak individuals to steer away from areas already occupied by strong individuals.

## Conclusions

The effect of a losing experience was only apparent in dominants that were facing an unfamiliar opponent, according with our hypothesis. In subordinates, however, opponent familiarity and contest experience affected behavior at different stages of a contest, and neither influence depended on the other. The reasons for this difference are not clear, but they could arise from uncertainty remaining even in contests with familiar opponents, from differences between dominants’ and subordinates’ responsiveness to contest experience or from the different behavioral measures that we had to adopt. Furthermore, the number of attacks that the dominants delivered to the subordinates in Contest 2 after they had retreated depended only on the aggressiveness of the subordinates: dominants attacked aggressive subordinates more often. Overall, these results show that information derived from opponent familiarity and the outcomes of previous contests influence the contest decisions of both subordinates and dominants. The different ways in which subordinates and dominants integrate information from these different sources warrants further investigation.

## Materials and methods

### Study organism

*Kryptolebias marmoratus* is an internally self-fertilizing hermaphroditic fish living in mangrove swamps from coastal regions of Brazil and eastern Central America, throughout the Caribbean to central Florida [47]. Natural populations consist mainly of isogenic homozygous hermaphrodites with < 1% males [48]. This fish is often found in intermittently dry shallow, stagnant pools, in crab burrows and inside/under logs and mangrove and leaf litter - intermittently dry microhabitats with adverse water quality conditions [46]. *K. marmoratus* has a number of morphological and physiological adaptations that enhance its ability to emerse, which allows the fish to escape poor water conditions and intra-specific aggression [46,49,50]. The fish does not exhibit schooling behaviour, but hides under cover and behaves aggressively in both the field and the laboratory [50-52]. In the field, the chasing of a smaller individual by a larger individual frequently results in the emersion of the smaller individual [50]. In the laboratory, when provided with small pieces of shelters/covers, one fish usually occupies one refuge, especially larger fish which tend to be territorial [52].

The fish is capable of producing fertilized eggs all year round, does not have obvious oviposition cycles [46] and laboratory fish usually start to lay fertilized eggs 3 to 6 months after hatching [53,54]. This study used F6 to F11 generations of five isogenic lineages of *K. marmoratus* originally collected by Dr. D. Scott Taylor from various locations (DAN2K: Dangriga, Belize; HON9: Utila, Honduras; RHL: San Salvador, Bahamas; SLC8E: St. Lucie County, FL, USA; VOL: Volusia County, Florida, USA). Because the fish do not exhibit grouping behavior, behave aggressively toward each other and are carnivorous and would devour each other [51], they were separated into individual containers within a month of hatching (but see [55] for a review of possible effects of isolation on fish aggression). They were placed in individual translucent polypropylene containers (13 × 13 × 9 cm) filled with 550 ml 25 ppt synthetic sea water (Instant Ocean™ powder) at the National Taiwan Normal University, and given a unique identification code. Fish were kept at 25 ± 2°C on a 14:10-h photoperiod and fed newly hatched brine shrimp (*Artemia*) nauplii daily. Containers were cleaned and water replaced every two weeks. All fish used in this study were at least 8 months old, larger than 20 mm in standard length (SL; from the tip of the snout to the caudal peduncle), and had been re-isolated for at least one month after use in previous studies as a precaution to avoid over-using them.

Experiments were conducted in accordance with National Taiwan Normal University Animal Care and Use Committee permit #99034.

### Experimental procedures

We divided the fish into size-matched (difference in SL ≤ 1 mm) pairs. The two individuals of a pair were also matched for their lineage (both individuals belonged to same lineage) and the outcome of their last contest prior to this study (both previous winners or both previous losers). The UF treatment required switching opponents between different pairs for Contest 2, requiring two pairs (same lineage and last outcome and difference in mean pair SL < 1 mm) to be tested simultaneously. We used this experimental regime for all six treatments. On Day 0 afternoon (roughly 1700 h), we marked the four fish of the two pairs by breaking the non-vascular thin membrane between the two soft rays in the dorsal fin, the pelvic fin or the upper or lower margins of the caudal fin (randomly assigned) with a needle. Immediately after marking, fish were replaced in their maintenance containers, and fed small amounts of newly hatched brine shrimp. All fish resumed regular feeding behavior within 5 s. Marking did not cause bleeding or observable adverse effects upon the fishes’ health or behavior. The membrane usually grows back completely in 3 days.

The experimental procedures consisted of six steps (Familiarization 1, Contest 1, Familiarization 2, Experience treatment, Familiarity treatment and Contest 2; Figure 1) and are explained in detail below.

### Familiarization 1

For both pairs, the two fish of a pair were then placed into the two similar-sized compartments (randomly determined) of a standard aquarium (12 × 8 × 20 cm, containing 2 cm of gravel and water filled up to 12 cm in height). The two compartments were separated by an opaque and a clear nylon-mesh partition (back-to-back) inserted in the middle of the aquarium. These partitions prevented the two individuals of a pair from physical and visual interaction, but did not prevent the exchange of chemical (olfactory) cues as water flowed between the two compartments.

On Day1 in the morning (0800 h), after approximately 15 h acclimatization, we removed the opaque partitions so that the two individuals of each of the two pairs could interact through the clear nylon-mesh partitions. This familiarization period lasted for two hours, and provided an opportunity for the two pair-mates to assess each other visually.

### Contest 1

After Familiarization 1, we removed the clear mesh partitions (1000 h) to allow the two individuals of both pairs to fight until the contests were resolved with clear winners and losers (see the section ‘Contest behavior’ below). The winner and the loser of a contest pair from Contest 1 are referred to as the dominant and the subordinate hereafter. The mean (± SE) time period for Contest 1 to resolve was 200.4 ± 24.2s. The two pairs resulted in two dominants and two subordinates.

### Familiarization 2

After Contest 1, the clear nylon-mesh partitions were re-inserted to prevent the dominants from chasing or attacking the subordinates but to allow more time for exchanges of chemical or visual signals between the two individuals in each pair. This second familiarization period also lasted for two hours.

### Experience treatment

The four individuals of the two pairs were transferred to four different standard aquaria to receive their pre-assigned contest experiences after Familiarization 2. (See the section ‘Providing a winning, losing or no-contest experience’ below.) Upon completion of the experience training, the fish were replaced in their maintenance containers, and fed newly hatched brine shrimp.

### Familiarity treatment

One hour after being fed, the four fish were paired up again according to their pre-designated familiarity treatment; a subordinate fish was paired either with the dominant fish that it had lost to in Contest 1 (familiar opponent treatment) or with the dominant fish from the other pair (unfamiliar opponent treatment). The two individuals of each of these pairs were placed one in each of the two similar-sized compartments (randomly assigned) of a new standard aquarium separated by an opaque partition and left to acclimatize overnight.

### Contest 2

On Day 2 morning (1000 h), the opaque partitions of the standard aquaria were removed to allow the pairs to fight until the contests (Contest 2) were resolved with clear winners and losers. The mean (± SE) time period for Contest 2 to resolve was 79.6 (± 9.2) s. After a 5-min status-confirmation period (see the section ‘Contest behavior’ below) the opaque partitions were reinserted to separate the contestants, and the fish returned to their maintenance containers and fed with newly hatched brine shrimp.

A total of 240 size-matched pairs (480 individuals) of fish were used for this study, evenly distributed over the six treatment × five lineage combinations.

### Contest behavior

The behaviors of the subordinate and dominant individuals in Contest 2s were recorded for the statistical analyses performed in this study. Please refer to previous studies of *K. marmoratus* for detailed descriptions of its contest behavior [22,27]. After the partition was removed, the fish usually oriented and moved toward each other, often with gill cover erected (opercular display). After a few bouts of mutual displays, if neither contestant retreated, one of the contestants would launch a first attack by swimming rapidly towards and pushing against or biting its opponent. The fish that received the first attack either retreated or responded with attacks. The individual that first retreated from its opponent’s displays/attacks for 5 min (status-confirmation period) without retaliating was the loser and its opponent the winner. During this period, losers were able to flip out of water and stick to the side of the aquarium to avoid post-contest harassment from the winners (“emersion” behavior described in “Study Organism”). All contests were video-taped and monitored by an observer siting behind the camcorder. The behavioral data were later transcribed from the recordings by experimenters who had no knowledge of the treatments to which the experimental fish had been assigned.

### Providing a winning, losing or no-contest experience

To ensure that fish received their pre-designated losing (or winning) experience, we fought them against much larger (smaller) fish (difference in SL > 2 mm) that had won (lost) several fights against similar-sized opponents. The experience training was staged by placing an experimental fish in one of the two similar-sized compartments (randomly assigned) of a standard aquarium divided by an opaque partition and the larger (smaller) trainer fish in the other compartment. After 15-min acclimatization, the partition was removed to allow the fish to interact. A losing experience was completed when the experimental fish retreated from a display/attack by the larger trainer fish and quickly swam away. Of the 80 dominants assigned to receive a losing experience, 23 retreated immediately after being attacked by their larger trainers and 57 retreated after some period (mean ± SE = 26.9 ± 5.6 s) of mutual physical interactions with their larger trainers. A winning experience was completed when the smaller trainer fish retreated from the experimental individual’s display/attack and quickly swam away. As all the small trainers retreated after this display/attack, the experimental fish acquired their winning experiences without mutual physical interaction. Upon the completion of the experience training, the partition was re-inserted to separate the two fish. Fish assigned to receive a no-contest experience were treated exactly as above, with procedures synchronized with those assigned losing or winning experience, except that there was no opponent in the other compartment.

### Statistical analyses

We used generalized linear models (binomial distribution with a logit link function) to examine whether and if so how familiarity and contest experience jointly affected the most aggressive behavior the subordinates and the dominants exhibited in Contest 2. When analyzing subordinates’ behavior, we included dominants’ behavior (not attacking directly or attacking directly) in the models to account for any possible influence it might have on the subordinates’ contest behaviors. And, when analyzing the dominant’s behavior, we included their subordinate opponents’ most aggressive behaviors (none, displaying or attacking) in the model to account for any influence it might have. We further used a generalized linear model (binomial distribution with a logit link function) to examine the influences of familiarity and contest experience on contest outcome (the likelihood of the subordinates winning Contest 2). For these generalized linear models, we used contrast analyses to evaluate the differences between different levels of treatment and interaction effects.

For the subset of Contest 2s won by dominants, we used a general linear model to examine the effect of familiarity and contest experience on the number of post-retreat attacks (square root transformed) that the dominants delivered to the subordinates after they had retreated (i.e., the number of attacks delivered in the 5-min status-confirmation period). We used an F test to examine the residuals from this model (normally distributed; Shapiro-Wilk test, *W* = 0.977, *P* = 0.141) and test whether the number of post-retreat attacks delivered by the dominant winners differed significantly between subordinates that exhibited different degrees of aggressiveness toward them.

We included body size (SL) and lineage in all regression models to account for their influence. SAS Enterprise Guide 5.1 (SAS Institute Inc., Cary, NC, USA) was used for the statistical analyses.
